# A randomized phase 2 trial of nintedanib and low-dose cytarabine in elderly patients with acute myeloid leukemia ineligible for intensive chemotherapy

**DOI:** 10.1007/s00277-022-05025-0

**Published:** 2022-11-18

**Authors:** Andrew F. Berdel, Raphael Koch, Joachim Gerss, Marcus Hentrich, Rudolf Peceny, Tobias Bartscht, Björn Steffen, Marina Bischoff, Karsten Spiekermann, Linus Angenendt, Jan-Henrik Mikesch, Tobias Kewitz, Trude Butterfass-Bahloul, Hubert Serve, Georg Lenz, Wolfgang E. Berdel, Utz Krug, Christoph Schliemann

**Affiliations:** 1 Department of Medicine A, University Hospital Münster, Albert-Schweitzer-Campus 1, 48149 Münster, Germany; 2 Institute of Biostatistics and Clinical Research, University of Münster, Münster, Germany; 3 Department of Hematology and Oncology, Red Cross Hospital, Munich, Germany; 4 Department of Oncology, Hematology and Stem Cell Transplantation, Klinikum Osnabrück, Osnabrück, Germany; 5 Department of Medicine I, University Hospital Lübeck, Lübeck, Germany; 6 Department of Medicine II, University Hospital Frankfurt, Frankfurt, Germany; 7 Department of Hematology and Oncology, Klinikum Idar-Oberstein, Idar-Oberstein, Germany; 8 Department of Medicine III, University Hospital Munich, Ludwig-Maximilians-University, Munich, Germany; 9 Centre for Clinical Trials, University of Münster, Münster, Germany; 10 Department of Medicine, III, Hospital Leverkusen, Leverkusen, Germany

**Keywords:** Acute myeloid leukemia, Nintedanib, Low-dose cytarabine, Angiogenesis

## Abstract

We investigated the safety and efficacy of nintedanib added to low-dose cytarabine (LDAC) in a phase 1/2 study in patients 60 years or older with newly diagnosed or relapsed/refractory (r/r) AML ineligible for intensive chemotherapy. The results of the dose-finding phase 1 part have been previously published. Patients were randomized 1:1 to LDAC plus nintedanib or LDAC plus placebo stratified by AML status (newly diagnosed vs r/r). LDAC was applied subcutaneously at 20 mg twice daily on days 1 to 10. Nintedanib/placebo was orally administered twice daily on days 1 to 28 in 28-day cycles. The primary endpoint was overall survival (OS). Between 05/2017 and 09/2019, 31 patients were randomized and 30 were treated, before the study was terminated prematurely due to slow recruitment. Median (range) age of patients was 76 (60–84) years. Twenty-two patients (73%) had r/r AML. Median OS in patients treated with LDAC and nintedanib was 3.4 months, compared with 3.6 months in those treated in the placebo arm, with a HR adjusted for AML status of 1.19 (corresponding confirmatory adjusted 95% CI, 0.55–2.56; univariate log-rank *P* = 0.96). In the 22 patients with r/r AML, median OS was 3.0 months in the nintedanib and 3.6 months in the placebo arm (*P* = 0.36). One patient in the nintedanib and two patients in the placebo arm achieved a CR and entered maintenance treatment. Nintedanib showed no superior therapeutic activity over placebo when added to LDAC in elderly AML patients considered unfit for intensive chemotherapy. The trial was registered at clinicaltrials.gov NCT01488344.

**Supplementary Information:**

The online version contains supplementary material available at 10.1007/s00277-022-05025-0.

## Introduction

Despite recent advances, effective and well-tolerable treatment options for patients with acute myeloid leukemia (AML) who are ineligible to receive intensive chemotherapy remain limited. Hypomethylating agents (HMA; e.g., azacitidine and decitabine) and low-dose cytarabine (LDAC) are increasingly being used as backbones for combination therapies with novel drugs. Venetoclax and glasdegib are two examples of molecularly targeted combination partners which have provided improved outcomes compared to HMA or LDAC monotherapies, but overall survival (OS) remains modest [1–3].

Nintedanib is a potent, orally available, small molecule triple tyrosine kinase inhibitor targeting vascular endothelial growth factor (VEGF) receptor VEGFR-1/-2/-3, fibroblast growth factor (FGF) receptor FGFR-1/-2/-3 and platelet derived growth factor (PDGF) receptor PDGFR-α/-β signaling [4]. All three pathways have been shown to be involved in the pathophysiology of AML. Produced by leukemic cells, VEGF, FGF, and PDGF act in a paracrine fashion on the bone marrow vasculature and stroma, thereby promoting leukemia cell support, growth and survival [5, 6]. Bone marrow microvessel formation is increased in AML, normalized in complete remission (CR) [7], and associated with inferior prognosis [8]. VEGF production by leukemic blasts and increased VEGF serum levels have negative prognostic impact in AML [9, 10]. Furthermore, all these ligand-receptor pairs are functional in autocrine signaling circuits, in which leukemia-derived growth factors directly support the growth of leukemic cells expressing the cognate surface receptors [11–17]. These observations argue for simultaneous inhibition of both auto- and paracrine loops for effective disease control. In addition, nintedanib has activity against Src family members and FLT3 [4]. In vitro, nintedanib displayed potent growth inhibitory and proapoptotic effects against various myeloid leukemia cell lines in the nanomolar range and increased the anti-leukemic activity of cytarabine [18].

In order to translate these preclinical findings, we investigated the safety and efficacy of nintedanib added to LDAC in a phase 1/2 study in patients 60 years or older with untreated or relapsed/refractory (r/r) AML ineligible for intensive chemotherapy. The results of the dose-finding phase 1 part have been previously published and suggested a continuation of the trial with a phase 2 recommended dose (P2RD) of 200 mg nintedanib twice daily in combination with LDAC [19]. Here, we report the findings of the randomized, double-blind, placebo-controlled phase 2 part of the study.

## Patients and methods

### Phase 2 study design and patients

The trial was registered at www.clinicaltrials.gov (identifier NCT01488344) and EudraCT (2011-001086-41). In accordance with a request by the Ethics Committee, the phase 2 part was modified into a randomized, double-blind, placebo-controlled design. The study was approved by the joined Ethics Committee of the Physicians Chamber of Westphalia-Lippe and the University of Muenster, Germany, and conducted in accordance with the Declaration of Helsinki and the International Conference on Harmonization Guideline for Good Clinical Practice across 10 sites in Germany.

The primary objective was to evaluate whether nintedanib, when combined with LDAC, improved overall survival (OS) of patients with previously untreated or r/r AML compared to LDAC plus placebo. OS was defined as the time interval from day one of study treatment to the day of death. For a patient who was not known to have died by the end of follow-up, observation of OS was censored on the date the patient was last known to be alive. Secondary and exploratory objectives were complete remission (CR) rate, overall response rate consisting of CR, CR with incomplete hematological recovery (CRi) and CR with incomplete platelet recovery (CRp) by International Working Group (IWG) criteria [20], relapse-free survival in responders, time to response, response and OS by cytogenetics and *FLT3* mutational status, safety and toxicity (CTCAE, version 4.03), and biomarkers.

Patients 60 years or older with newly diagnosed or r/r AML who were ineligible for intensive chemotherapy were enrolled. Ineligibility for intensive therapy was based on investigator assessment of AML characteristics (e.g., genetics, type of AML) and patient characteristics (e.g., age, comorbidities, performance status). Exclusion criteria were acute promyelocytic leukemia, bone marrow blast count of less than 30% (at the time of study onset qualifying for azacitidine therapy), inadequate liver (ALT, AST ≥ 1.5 × ULN if not due to AML infiltration) and renal (creatinine clearance < 45 mL/min) function, central nervous system (CNS) involvement of AML, chronic or active hepatitis C, uncontrolled hypertension (> 160 mmHg systolic, > 95 mmHg diastolic), larger trauma or surgery within the last 4 weeks, chronic wound healing problems including bone fractures, uncontrolled infections, previous therapy with tyrosine kinase inhibitors or angiogenesis inhibitors, specific contraindications against cytarabine or nintedanib, relevant other diseases possibly influencing study endpoints, and participation in another interventional clinical trial within 4 weeks. All patients provided written informed consent prior to trial enrollment.

Patients were randomized in a 1:1 ratio to receive either LDAC plus nintedanib or LDAC plus placebo, stratified by AML status (newly diagnosed versus r/r).

### Treatment

LDAC was applied subcutaneously at 20 mg twice daily on days 1 to 10 in 28-day cycles. Nintedanib or placebo tablets identical in appearance (Boehringer Ingelheim Pharma GmbH & Co. KG, Biberach an der Riss, Germany) were orally applied at 200 mg twice daily on days 1 to 28. Dose reductions to 150 mg or 100 mg nintedanib/placebo twice daily were allowed. As a general rule, non-hematological grade 3 toxicities should be followed by permanent dose reduction off nintedanib/placebo, unless effectively controlled with supportive therapy. Specific rules applied for gastrointestinal toxicities and liver enzyme elevations. Patients could continue treatment for up to 6 cycles of LDAC plus nintedanib/placebo, disease progression or relapse, intolerance, or request of treatment discontinuation by patient or investigator. Patients without disease progression after 6 cycles could enter oral maintenance treatment with nintedanib/placebo (up to 12 months from start of therapy).

### Study assessments and statistical analyses

Statistical analyses were performed according to the principles of the ICH-guideline E9 “Statistical Principles for Clinical Trials.” The primary outcome was OS. A sample size of 100 patients was initially planned to provide a sufficiently precise 95% CI for the hazard ratio (HR, nintedanib vs placebo) of OS. With the initial assumptions of exponential-distributed OS (median OS 176 days in the nintedanib arm vs 110 days in the placebo arm, HR 0.625, 80 events in total), the probability to observe a confirmatory 95% CI for the HR with half-width ≤ 0.34 was 80%. Patients were randomly assigned to treatment of either LDAC plus nintedanib or LDAC plus placebo in a 1:1 ratio. Randomization was stratified by AML status (newly diagnosed and r/r AML). A block randomization was used with a block length of 4.

Data base closure was on July 27, 2021. Measurements were summarized by descriptive statistics. Statistical analyses for time-to-event endpoints included the Kaplan–Meier estimator, log-rank test, Cox proportional hazard regression model, and competing risk approach. In the primary analysis, a confirmatory two-sided 95% confidence interval (CI) for the HR of nintedanib vs placebo adjusted for AML status was established in the full-analysis-set using the intention-to-treat (ITT) principle. The Cox model included OS as dependent variable and the main effect of the AML status variable and the treatment arm variable as covariates. The primary analysis was calculated by a 95% CI for the HR by using a Cox regression with treatment arm and AML status (newly diagnosed vs r/r) as independent variables. For sensitivity analysis, the unadjusted 95% CI was computed using a Cox model including the treatment arm variable as the only covariate and both arms were compared using a log-rank test. In addition, analyses were repeated in the per-protocol collective (results not shown).

For safety analyses, patients were analyzed according to the received study treatment (as treated). All patients who received any dose of either nintedanib or placebo were included in the safety and efficacy analyses. Adverse events (AE) and laboratory abnormalities were based on Common Terminology Criteria for Adverse Events (CTCAE, version 4.03) and were coded according to Medical Dictionary for Regulatory Activities (MedDRA, version 21.1). AEs were summarized by type, frequency, severity, relatedness, and seriousness. The numbers of patients who developed toxicities within a system organ class were compared between the two groups using Fisher’s exact tests. All *P* values and CIs (beyond the primary) were intended to be exploratory, not confirmatory. Therefore, no adjustment for multiplicity was made. Exploratory two-sided *P* values ≤ 0.05 were considered to be statistically noticeable. All analyses were performed using SAS software, version 9.4 TS1M7, of the SAS System for Windows.

## Results

**Fig. 1:**
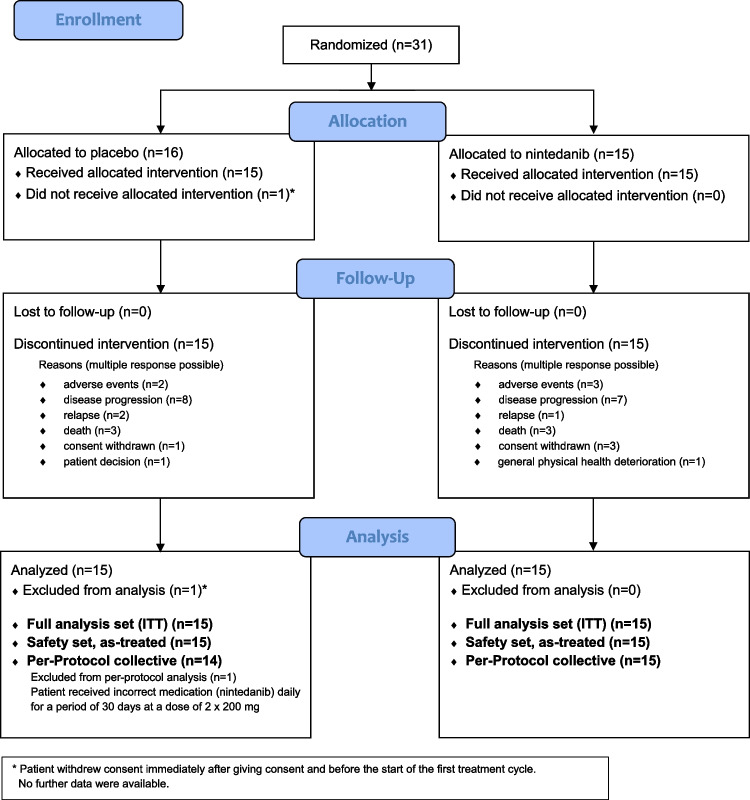
CONSORT flowchart. *ITT*, intention to treat

**Fig. 2:**
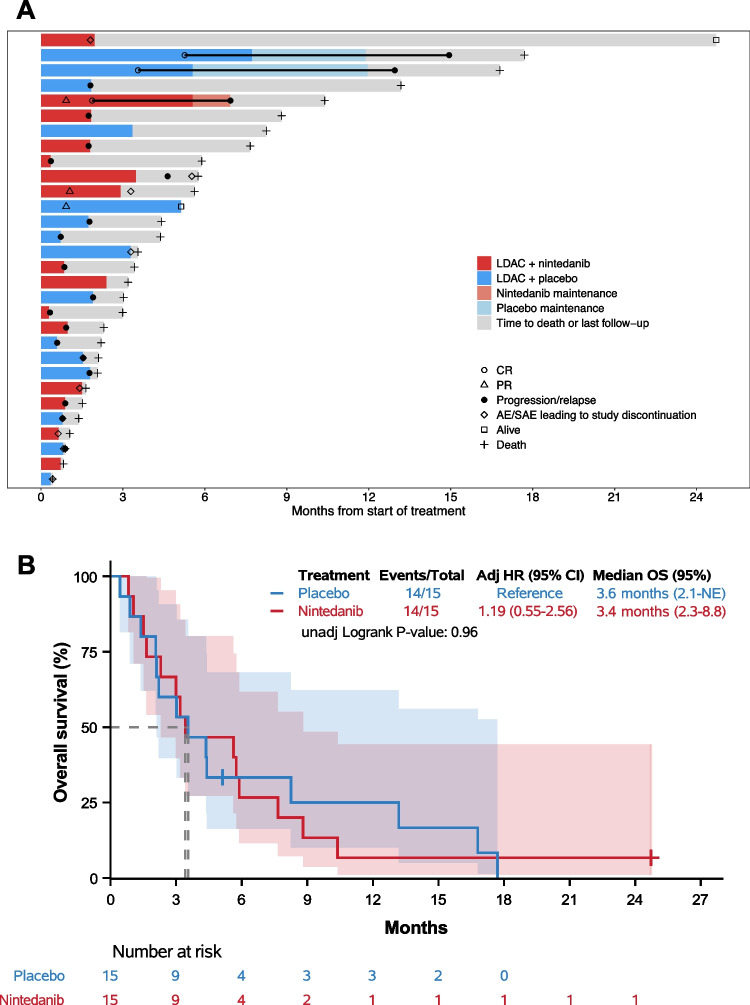
Patient outcome and overall survival. (**A**) Swimmer plot of individual treatment courses and outcome in all 30 patients. Treatment allocation is color-coded. (**B**) Kaplan–Meier estimates of OS of all 30 patients treated in the study. Dashed lines mark the median survival time. The transparent areas represent the pointwise 95% CI (log-transformed) of the Kaplan–Meier estimates. Abbreviations: *HR*, hazard ratio; *NE*, not estimable*; adj*, adjusted*; unadj*, unadjusted*; OS*, overall survival

On December 31, 2019, study enrollment was prematurely stopped due to slow recruitment. Between May 2017 and September 2019, 31 patients were enrolled and randomized. One patient withdrew consent before initiation of study treatment and was therefore excluded. Thirty patients (15 in each study arm) received at least one dose of nintedanib/placebo and were included in the safety and efficacy analyses. Details are given in the CONSORT flowchart (Fig. 1) and each patient’s individual course of treatment is depicted in the swimmer plot (Fig. 2A).

 Patient demographics and baseline disease characteristics are shown in Table 1. Twenty-two patients (73%) had r/r AML. No relevant imbalances between the randomized arms were observed with the exception that patients with r/r AML in the placebo arm entered the study after slightly more lines of previous therapy compared to patients in the nintedanib arm. Median (range) age of all patients treated was 76 (60–84) years. Thirty percent (9/30) of patients had adverse risk genetics according to European LeukemiaNet (ELN) 2010 definitions and 33% (10/30) had secondary or therapy-related AML (s-AML/t-AML).

**Table 1:** Patient characteristics

Variables	Total	Nintedanib	Placebo
N	30	15	15
Age, years			
Median (range)	76 (60–84)	77 (60–84)	75 (61–81)
Sex, no (%)			
Male	15 (50)	7 (47)	8 (53)
Female	15 (50)	8 (53)	7 (47)
ECOG PS, no (%)			
0	2 (7)	2 (13)	0
1	18 (60)	8 (53)	10 (67)
2	9 (30)	4 (27)	5 (33)
3	1 (3)	1 (7)	0
AML status, no (%)			
Newly diagnosed	8 (27)	4 (27)	4 (27)
Relapsed or refractory	22 (73)	11 (73)	11 (73)
AML type, no (%)			
de novo	7 (23)	4 (27)	3 (20)
s-AML	9 (30)	5 (33)	4 (27)
t-AML	1 (3)	1 (7)	0
Unknown	13 (43)	5 (33)	8 (53)
WBC, G/l			
Median (range)	5.18 (0.83–78.5)	5.61 (1.06–77.13)	3.88 (0.83–78.55)
Blasts in peripheral blood, %			
Median (range)	17.5 (0–96)	20.5 (0–68)	16.5 (0–96)
Blasts in bone marrow, %			
Median (range)	57 (13–95)	67 (13–95)	50 (22–95)
ELN 2010 risk, no (%)			
Favorable	6 (20)	4 (27)	2 (13)
Intermediate I	8 (27)	4 (27)	4 (27)
Intermediate II	4 (13)	3 (20)	1 (7)
Adverse	9 (30)	4 (27)	5 (33)
Unknown/not determinable	3 (10)	0	3 (20)
*FLT3***-**ITD, no (%)			
Present	3 (10)	3 (20)	0
Absent	10 (33)	5 (33)	5 (33)
Unknown	17 (57)	7 (47)	10 (67)
*NPM1*, no (%)			
Mutated	7 (23)	4 (27)	3 (20)
Wild type	11 (37)	6 (40)	5 (33)
Unknown	12 (40)	5 (33)	7 (47)
Prior treatments, no (%)			
0	8 (27)	4 (27)	4 (27)
1	9 (30)	5 (33)	4 (27)
2	5 (17)	3 (20)	2 (13)
≥ 3	5 (17)	1 (7)	4 (27)
Unknown	3 (10)	2 (13)	1 (7)

Abbreviations: *ECOG*, Eastern Cooperative Oncology Group; *PS*, performance status; *AML*, acute myeloid leukemia; *s-AML*, secondary AML; *t-AML*, therapy-related AML; *ELN*, European LeukemiaNet; *FLT3-ITD,* internal tandem duplication of the FLT3 gene; *NPM1*, nucleophosmin-1; *WBC*, white blood cells

Median (range) duration of treatment was 46 (9–211) days in the nintedanib and 55 (11–362) days in the placebo arm. In both arms, the median (range) number of treatment cycles initiated was 2 (1–6). One and two patients entered maintenance therapy in the nintedanib and placebo arm, respectively. For each patient, multiple reasons for study discontinuation could be reported. Primary reasons were: AML progression (nintedanib: 7; placebo: 8), death (nintedanib. 3; placebo: 3), AE (nintedanib: 3; placebo: 2), consent revoked (nintedanib: 3; placebo: 1), relapse (nintedanib: 1; placebo: 2), patient decision (nintedanib: 0; placebo: 1) and general physical health deterioration (nintedanib: 1; placebo: 0).

### Efficacy

At the time of analysis, 28 of 30 patients had died. One patient was lost to follow-up after 5.1 months, and the one remaining living patient was followed for 24.7 months (Fig. 2A). No difference in OS between the treatment arms could be detected. The adjusted HR for nintedanib vs placebo was 1.19 and the corresponding confirmatory 95% CI was 0.55–2.56 (Cox regression Wald *P* = 0.66). The non-stratified univariate comparison of OS between both treatment arms resulted in a HR of 1.02 (95% CI 0.48–2.15, log-rank *P* = 0.96). Median OS in patients treated with LDAC and nintedanib was 3.4 months, compared with 3.6 months in those treated in the placebo arm. Six-month OS was 27% in the nintedanib and 33% in the placebo arm (Fig. 2B).

In the 22 patients enrolled into the study with r/r AML, median OS was 3.0 months in the nintedanib and 3.6 months in the placebo arm, respectively (11/11 vs 10/11 events, HR 1.54, 95% CI, 0.61–3.86; log-rank *P* = 0.36; Figure S1A). Likewise, no noticeable difference in OS in the subgroup of patients with newly diagnosed AML could be detected between the two treatments (8.2 vs 5.6 months in the nintedanib and placebo arm, respectively; HR 0.55, 95% CI, 0.12–2.51; log-rank *P* = 0.44; Figure S1B). Results of additional sensitivity analyses in the per-protocol collective led to similar results (data not shown). Further time-to-event or subgroup analyses were not interpretable due to the small number of patients.

Three out of all 30 patients (10%) achieved a CR, 2 in the placebo and 1 in the nintedanib arm (Fig. 2A). No patient achieved any other overall response criterion (CRi/CRp) without subsequently achieving a CR. One CR was observed in a patient with newly diagnosed AML and favorable risk genetics in the placebo arm. Two CRs were observed in patients with r/r AML and intermediate risk genetics, one in the nintedanib and placebo arm, respectively (see Table S2 for further disease characteristics of responding patients). In addition, one patient in each arm achieved a partial remission (PR). Nine patients did not receive a formal post-baseline BM response assessment, 3 in the placebo arm and 6 in the nintedanib arm. Five of these were classified as having PD by the investigator and response was non-evaluable in 4 patients (Fig. 2A).

Due to the small number of responders, no statistics were computed for relapse-free survival (RFS), but all 3 responders experienced AML relapse after a remission duration of 5.1, 9.4, and 9.7 months. Likewise, no further subgroup analyses for molecularly defined subgroups were performed.

### Safety profile

All 30 patients receiving at least one dose of nintedanib/placebo were evaluated for safety. No AEs occurred before treatment start. A summary of AEs occurring in at least three patients (10%) and all serious AEs (SAE) are shown in Table 2. A complete list of all AEs is given in Table S1.

**Table 2:** Adverse events occurring in at least 10% of patients and all serious adverse events

	Nintedanib (*N*** = **15)		Placebo (*N*** = **15)	
Adverse events	Any CTCAE grade	CTCAE grade ≥ 3	Any CTCAE grade	CTCAE grade ≥ 3
*Preferred term MedDRA v21.1*	*Number of patients (percent)*			
Vomiting	8 (53)	1 (7)	0	0
Diarrhea	7 (47)	4 (27)	5 (33)	0
Nausea	7 (47)	1 (7)	4 (27)	0
Fatigue	5 (33)	3 (20)	2 (13)	1 (7)
Pyrexia	3 (20)	1 (7)	2 (13)	0
Febrile neutropenia	2 (13)	2 (13)	5 (33)	5 (33)
Abdominal pain	2 (13)	1 (7)	2 (13)	0
Pneumonia	2 (13)	2 (13)	2 (13)	2 (13)
Purpura	2 (13)	0	1 (7)	0
Cough	1 (7)	0	3 (20)	0
Epistaxis	1 (7)	0	3 (20)	0
Edema peripheral	1 (7)	0	2 (13)	0
Infection	1 (7)	0	2 (13)	1 (7)
Urinary tract infection	1 (7)	0	2 (13)	0
Headache	0	0	3 (20)	0
Pain in extremity	0	0	3 (20)	0
Serious Adverse Events	Any CTCAE grade	CTCAE grade ≥ 3	Any CTCAE grade	CTCAE grade ≥ 3
*Preferred term MedDRA v21.1*	*Number of patients (percent)*			
Febrile neutropenia	2 (13)	2 (13)	5 (33)	5 (33)
Pneumonia	2 (13)	2 (13)	2 (13)	2 (13)
Diarrhea	2 (13)	2 (13)	0	0
Neutropenic infection	1 (7)	1 (7)	1 (7)	1 (7)
Abdominal pain upper	1 (7)	1 (7)	0	0
Anal fissure	1 (7)	0	0	0
Circulatory collapse	1 (7)	0	0	0
Dyspnea	1 (7)	0	0	0
Fatigue	1 (7)	1 (7)	0	0
General physical condition decreased	1 (7)	1 (7)	0	0
Leukopenia	1 (7)	1 (7)	0	0
Lung infection	1 (7)	1 (7)	0	0
Nausea	1 (7)	1 (7)	0	0
Orthostatic hypotension	1 (7)	0	0	0
Pancytopenia	1 (7)	0	0	0
Pyrexia	1 (7)	1 (7)	0	0
Syncope	1 (7)	1 (7)	0	0
Vomiting	1 (7)	1 (7)	0	0
Disease progression	0	0	2 (13)	2 (13)
General physical health deterioration	0	0	2 (13)	1 (7)
Anemia	0	0	1 (7)	1 (7)
Cardiac failure	0	0	1 (7)	1 (7)
Febrile infection	0	0	1 (7)	1 (7)
Hematoma	0	0	1 (7)	1 (7)
Hemoglobin decreased	0	0	1 (7)	1 (7)
Infection	0	0	1 (7)	1 (7)
Refractoriness to platelet transfusion	0	0	1 (7)	1 (7)
Sepsis	0	0	1 (7)	1 (7)
Transfusion related complication	0	0	1 (7)	1 (7)

A total of 281 AEs were reported, 162 in the nintedanib arm (58%) and 119 in the placebo arm (42%). These were coded as 286 MedDRA codes (nintedanib: 165/286, 58%; placebo: 121/286, 42%). Ninety-nine AEs were deemed related to study treatment (nintedanib: 71/162 (44%); placebo: 28/119 (24%)). All patients had at least one AE with a median (range) frequency of 6.5 AEs (1–57). Most commonly, and in line with the known toxicity profile of nintedanib, patients experienced diarrhea (nintedanib: 7; placebo: 5), nausea (nintedanib: 7; placebo: 4) and vomiting (nintedanib: 8; placebo: 0). In 8 patients, AEs led to temporary treatment interruptions (nintedanib: 5; placebo: 3). AEs leading to study discontinuation were reported in 10 patients (nintedanib: 5 (4 infection-related, 1 diarrhea); placebo: 5 (2 disease progression, 1 infection-related, 1 infection-related with cardiac failure, 1 general physical health deterioration)).

A total of 45 SAEs occurred (nintedanib: 24; placebo: 21) in 21 patients (nintedanib: 9; placebo: 12), which were coded as 48 MedDRA codes. Thirteen SAEs were deemed related to study treatment (nintedanib: 9; placebo: 4). Most frequently, febrile neutropenia occurred in 7 patients (nintedanib: 2; placebo: 5) and pneumonia in 4 patients (nintedanib: 2; placebo: 2). SAEs led to temporary treatment interruption in 7 patients (nintedanib: 4; placebo: 3) and to study discontinuation in 9 patients (nintedanib: 4; placebo: 5). Nine patients died due to SAEs (nintedanib: 3 (all infection-related); placebo: 6 (3 infection-related issues, 2 disease progression and 1 general physical health deterioration)).

**Fig. 3:**
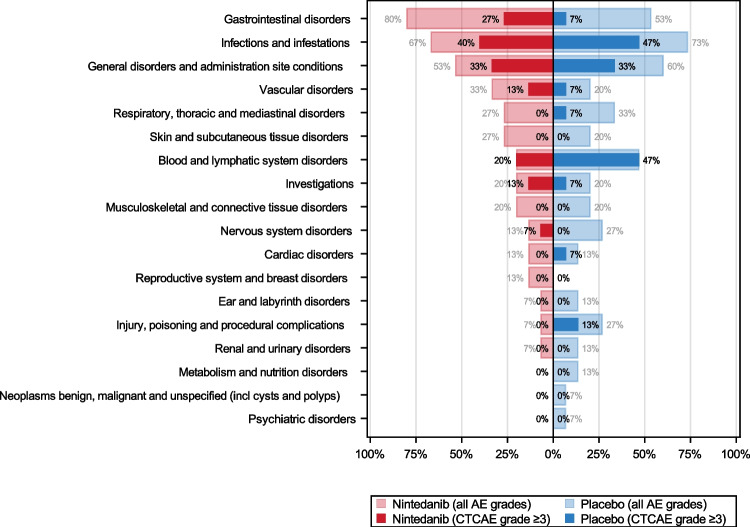
Adverse events. Butterfly plot displaying the percentage of patients with at least one AE or with at least one AE of CTCAE grade ≥ 3 by system organ class

No statistically noticeable differences (*P* ≤ 0.05, Fisher’s exact test) were observed between the two treatment arms in terms of number of patients with at least one AE by system organ class (Fig. 3, Figure S2). Slightly more patients had gastrointestinal disorders in the nintedanib group (12 vs 8, OR 3.35, 95% CI 0.55–26.35; *P* = 0.245), and more of those were CTCAE grade ≥ 3 events (4 vs 1, OR 4.84, 95% CI 0.4–268.23, *P* = 0.33).

## Discussion

Treatment of AML in elderly patients ineligible for intensive chemotherapy remains a significant challenge. This randomized, double-blind, placebo-controlled phase 2 trial was conducted to evaluate the efficacy and safety of nintedanib, a small molecule triple angiokinase inhibitor, in combination with LDAC in older patients with AML who were considered ineligible for intensive chemotherapy. The results of the dose-finding phase 1 part of the study had been published before and suggested a continuation of the trial with a phase 2 recommended dose (P2RD) of 200 mg nintedanib twice daily in combination with LDAC [19].

The study was stopped prematurely with approximately one-third of the patient number initially planned. Reasons for slow recruitment might have been competing trials in an identical patient cohort and increasing availabilities of alternative treatment options (e.g., venetoclax-based therapies).

While nintedanib plus LDAC continued to present a well manageable safety profile, the addition of nintedanib to LDAC was not associated with a superior OS compared to placebo and LDAC. Likewise, no signals for a beneficial effect of adding nintedanib were observed in the subgroup of patients who entered the study with r/r AML, whereas no conclusion could be made for the group of patients with previously untreated AML, due to low numbers. Response rates, time on treatment, 30-day mortality, and number of patients reaching maintenance therapy were also similar in both arms. Overall, the outcome in our study was comparable to outcomes reported in larger trials using LDAC monotherapy as a comparator [21, 22].

This disappointing result is in line with previous observations on the lack of efficacy of other anti-angiogenic drugs in AML, such as the VEGF antibody bevacizumab when given alone [23] or in combination [24]. However, other tyrosine kinase inhibitors clearly added to the therapeutic armory against AML. The multi-kinase pathway inhibitor sorafenib, active against RAS/RAF, c-kit, VEGFR, PDGFR, and FLT3, was previously investigated in addition to chemotherapy induction and showed no superior effect in AML patients older than 60 years [25], but improved event-free survival in younger patients, albeit with increased toxicity [26]. Furthermore, sorafenib maintenance therapy reduced the risk of relapse and death after allogeneic stem cell transplantation in *FLT3*-ITD-positive AML in the SORMAIN trial [27]. The first tyrosine kinase inhibitor demonstrating a significant improvement of OS when added to first-line induction chemotherapy was midostaurin in patients with *FLT3*-mutant AML [28]. Similarly, the second-generation FLT3 inhibitors quizartinib and gilteritinib have been shown to improve OS when added to intensive chemotherapy [29] or as monotherapy in r/r *FLT3*-mutant AML as compared to standard salvage chemotherapy [30, 31]. Unfortunately, for nintedanib, we cannot draw any conclusion from this study in terms of efficacy in *FLT3*-mutant AML, due to the small number of *FLT3*-mutant cases.

In conclusion, the triple angiokinase inhibitor nintedanib, approved for the treatment of advanced non-small cell lung cancer and fibrotic interstitial lung disease, had a manageable safety profile consistent with prior data, but showed no superior therapeutic activity over placebo when added to LDAC in elderly AML patients considered unfit for intensive chemotherapy.

## Supplementary Information

Below is the link to the electronic supplementary material.

Supplementary file1 (PDF 439 KB)

## Data Availability

The datasets generated during and/or analyzed during the current study are available from the corresponding author on reasonable request.
