# Supplementary material for: A randomized phase 2 trial of nintedanib and low-dose cytarabine in elderly patients with acute myeloid leukemia ineligible for intensive chemotherapy
Source: Ann Hematol. 2022 Nov 18;102(1):63–72. doi: 10.1007/s00277-022-05025-0 (PMC9807538; doi:10.1007/s00277-022-05025-0)
Supplement: Supplementary file 1 — Supplementary file1 (PDF 439 KB) [file 277_2022_5025_MOESM1_ESM.pdf]

A Randomized Phase 2 Trial of Nintedanib and Low-dose Cytarabine in Elderly Patients with Acute Myeloid Leukemia Ineligible for Intensive Chemotherapy

Andrew F. Berdel et al.

Supplemental Material

Supplemental Figures

Figure S1. Overall survival in (A) r/r AML and (B) newly diagnosed AML patients. Dashed lines mark the median survival time. The transparent areas represent the pointwise 95% confidence intervals (log-transformed) of the Kaplan-Meier estimates. Abbreviations: *HR*, hazard ratio; *NE*, not estimable; *OS*, overall survival.

A

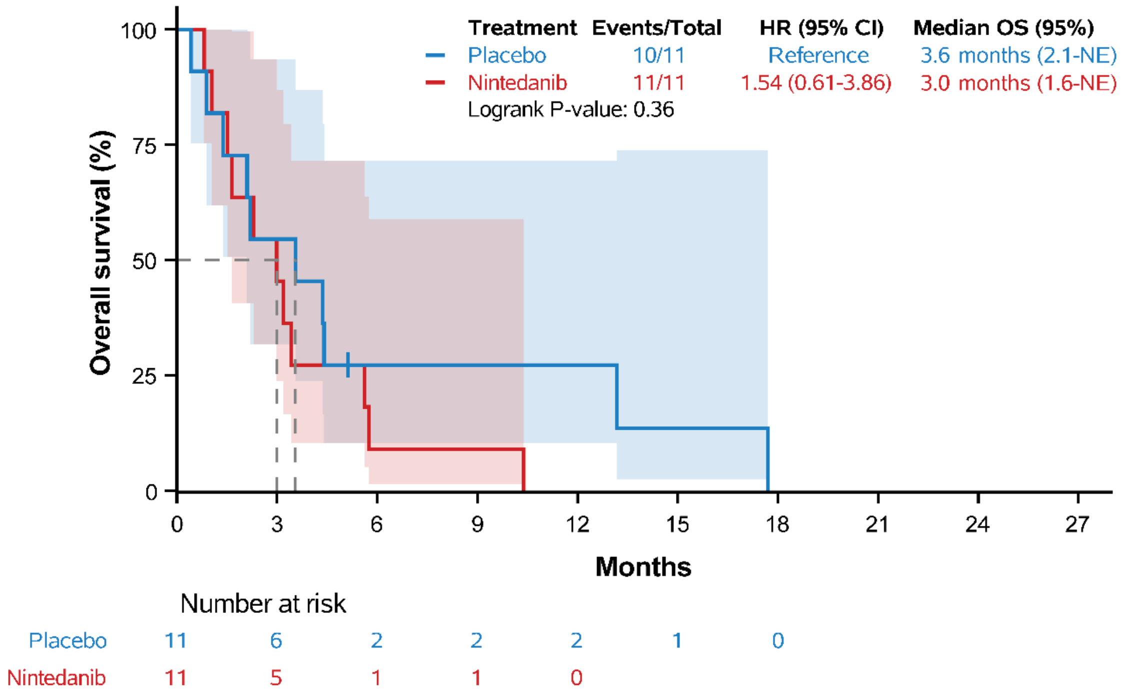

B

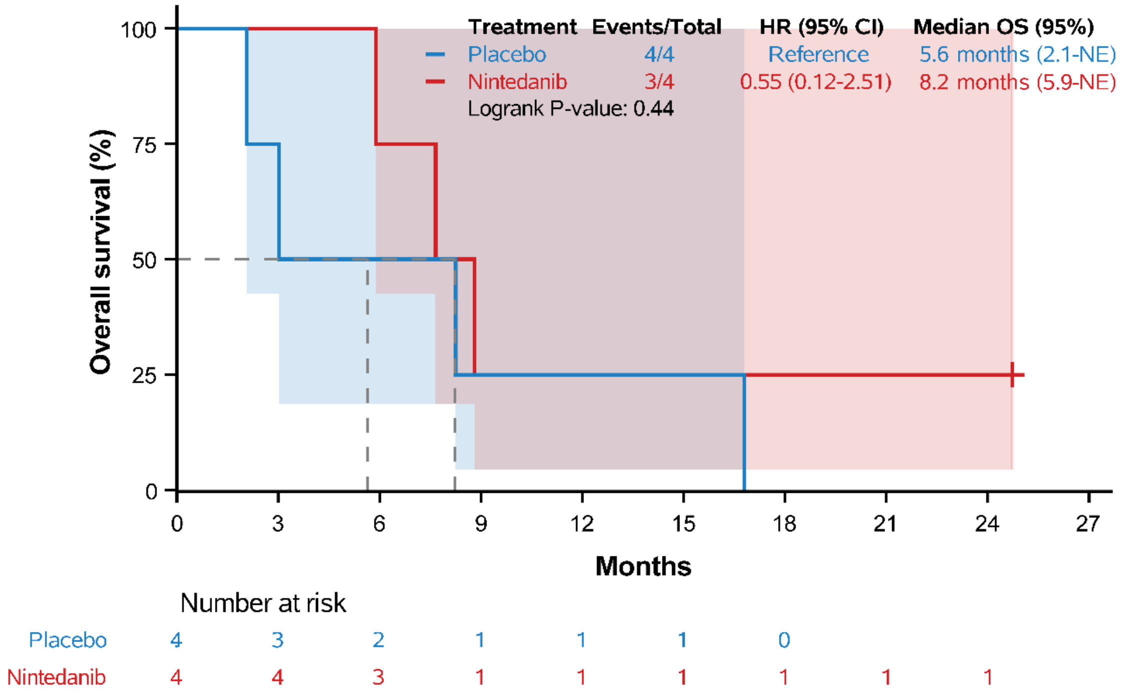

Figure S2. Forest plot of the patients with toxicities by system organ class (A: any CTCAE grade, B: CTCAE grade ≥ 3). Abbreviations: *OR*, odds ratio; *inf*, infinity.

A

Toxicity	Placebo Events/N (%)	Nintedanib Events/N (%)	OR (95% CI)	P Value*
Gastrointestinal disorders	8/15 (53.3%)	12/15 (80.0%)	3.35 (0.55, 26.35)	0.245
Reproductive system and breast disorders	0/15 (0.0%)	2/15 (13.3%)	2.54 (0.29, Inf)	0.483[2]
Vascular disorders	3/15 (20.0%)	5/15 (33.3%)	1.95 (0.29, 15.84)	0.682
Skin and subcutaneous tissue disorders	3/15 (20.0%)	4/15 (26.7%)	1.44 (0.19, 12.12)	1.000
Cardiac disorders	2/15 (13.3%)	2/15 (13.3%)	1.00 (0.06, 15.77)	1.000
Neoplasms benign, malignant and unspecified	1/15 (6.7%)	0/15 (0.0%)	1.00 (<0.01, 19.00)	1.000[1]
Psychiatric disorders	1/15 (6.7%)	0/15 (0.0%)	1.00 (<0.01, 19.00)	1.000[1]
Investigations	3/15 (20.0%)	3/15 (20.0%)	1.00 (0.11, 9.06)	1.000
Musculoskeletal and connective tissue disorders	3/15 (20.0%)	3/15 (20.0%)	1.00 (0.11, 9.06)	1.000
General disorders and administration site conditions	9/15 (60.0%)	8/15 (53.3%)	0.77 (0.14, 4.05)	1.000
Infections and infestations	11/15 (73.3%)	10/15 (66.7%)	0.74 (0.11, 4.55)	1.000
Respiratory, thoracic and mediastinal disorders	5/15 (33.3%)	4/15 (26.7%)	0.74 (0.11, 4.55)	1.000
Ear and labyrinth disorders	2/15 (13.3%)	1/15 (6.7%)	0.48 (0.01, 10.18)	1.000
Renal and urinary disorders	2/15 (13.3%)	1/15 (6.7%)	0.48 (0.01, 10.18)	1.000
Nervous system disorders	4/15 (26.7%)	2/15 (13.3%)	0.44 (0.03, 3.73)	0.651
Metabolism and nutrition disorders	2/15 (13.3%)	0/15 (0.0%)	0.39 (<0.01, 3.42)	0.483[1]
Blood and lymphatic system disorders	7/15 (46.7%)	3/15 (20.0%)	0.30 (0.04, 1.81)	0.245
Injury, poisoning and procedural complications	4/15 (26.7%)	1/15 (6.7%)	0.21 (0.00, 2.49)	0.330

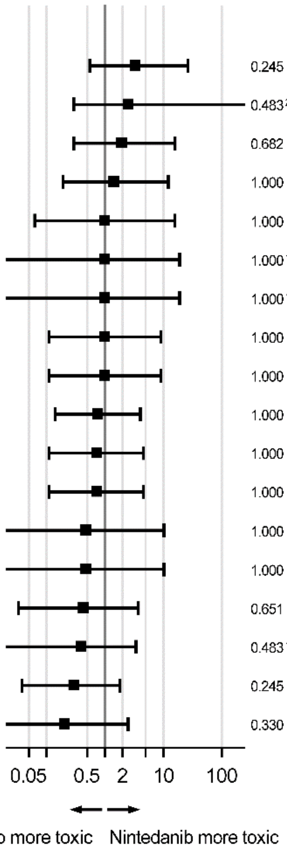

[1] upper one-sided p-value [2] lower one-sided p-value

* P-Values are from two-sided Fisher's exact tests.

B

Toxicity	Placebo Events/N (%)	Nintedanib Events/N (%)	OR (95% CI)	P Value*
Gastrointestinal disorders	1/15 (6.7%)	4/15 (26.7%)	4.84 (0.40, 268.23)	0.330
Investigations	1/15 (6.7%)	2/15 (13.3%)	2.10 (0.10, 135.99)	1.000
Vascular disorders	1/15 (6.7%)	2/15 (13.3%)	2.10 (0.10, 135.99)	1.000
Cardiac disorders	1/15 (6.7%)	0/15 (0.0%)	1.00 (<0.01, 19.00)	1.000[1]
Nervous system disorders	0/15 (0.0%)	1/15 (6.7%)	1.00 (0.05, Inf)	1.000[2]
Respiratory, thoracic and mediastinal disorders	1/15 (6.7%)	0/15 (0.0%)	1.00 (<0.01, 19.00)	1.000[1]
General disorders and administration site conditions	5/15 (33.3%)	5/15 (33.3%)	1.00 (0.17, 5.93)	1.000
Infections and infestations	7/15 (46.7%)	6/15 (40.0%)	0.77 (0.14, 4.05)	1.000
Injury, poisoning and procedural complications	2/15 (13.3%)	0/15 (0.0%)	0.39 (<0.01, 3.42)	0.483[1]
Blood and lymphatic system disorders	7/15 (46.7%)	3/15 (20.0%)	0.30 (0.04, 1.81)	0.245

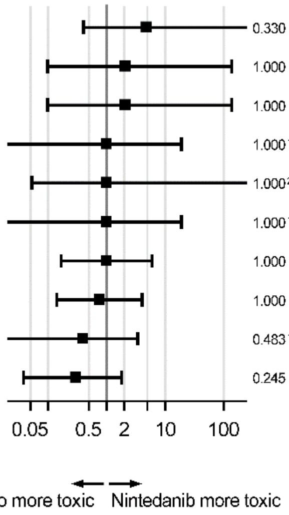

[1] upper one-sided p-value [2] lower one-sided p-value

* P-Values are from two-sided Fisher's exact tests.

Supplemental Tables

Table S1. Number of patients with adverse events.

	Nintedanib (N=15)		Placebo (N=15)	
**Adverse Events**	Any CTCAE grade	CTCAE grade ≥ 3	Any CTCAE grade	CTCAE grade ≥ 3
Preferred term MedDRA v21.1	*Number of patients (percent)*			
Vomiting	8 (53)	1 (7)	0	0
Diarrhoea	7 (47)	4 (27)	5 (33)	0
Nausea	7 (47)	1 (7)	4 (27)	0
Fatigue	5 (33)	3 (20)	2 (13)	1 (7)
Pyrexia	3 (20)	1 (7)	2 (13)	0
Febrile neutropenia	2 (14)	2 (13)	5 (33)	5 (33)
Abdominal pain	2 (13)	1 (7)	2 (13)	0
Pneumonia	2 (13)	2 (13)	2 (13)	2 (13)
Purpura	2 (13)	0	1 (7)	0
Dyspnoea	2 (13)	0	0	0
Cough	1 (7)	0	3 (20)	0
Epistaxis	1 (7)	0	3 (20)	0
Oedema peripheral	1 (7)	0	2 (13)	0
Infection	1 (7)	0	2 (13)	1 (7)
Urinary tract infection	1 (7)	0	2 (13)	0
Aphthous ulcer	1 (7)	0	1 (7)	0
Constipation	1 (7)	0	1 (7)	0
Haematoma	1 (7)	1 (7)	1 (7)	1 (7)
Lung infection	1 (7)	1 (7)	1 (7)	1 (7)
Mucosal inflammation	1 (7)	0	1 (7)	0
Neutropenic infection	1 (7)	1 (7)	1 (7)	1 (7)
Oedema	1 (7)	0	1 (7)	1 (7)
Pain	1 (7)	1 (7)	1 (7)	0
Pollakiuria	1 (7)	0	1 (7)	0
Vertigo	1 (7)	0	1 (7)	0
Abdominal pain upper	1 (7)	1 (7)	0	0
Abscess soft tissue	1 (7)	1 (7)	0	0
Alanine aminotransferase increased	1 (7)	1 (7)	0	0
Anal fissure	1 (7)	0	0	0
Arthralgia	1 (7)	0	0	0
Back pain	1 (7)	0	0	0
Bacterial disease carrier	1 (7)	0	0	0
Blister	1 (7)	0	0	0
Breast swelling	1 (7)	0	0	0
Circulatory collapse	1 (7)	0	0	0
Chest wall haematoma	1 (7)	0	0	0
Dizziness	1 (7)	0	0	0
Enterococcal infection	1 (7)	0	0	0
Fall	1 (7)	0	0	0
General physical condition decreased	1 (7)	1 (7)	0	0
Hypertension	1 (7)	1 (7)	0	0
Injection site reaction	1 (7)	0	0	0
Leukopenia	1 (7)	1 (7)	0	0
Lip infection	1 (7)	0	0	0
Liver function test increased	1 (7)	0	0	0
Mouth ulceration	1 (7)	0	0	0
Myalgia	1 (7)	0	0	0
Oral candidiasis	1 (7)	0	0	0
Orthostatic hypotension	1 (7)	0	0	0
Pancytopenia	1 (7)	0	0	0
Pathogen resistance	1 (7)	0	0	0
Pelvic infection	1 (7)	1 (7)	0	0

Pelvic pain	1 (7)	0	0	0
Peripheral swelling	1 (7)	0	0	0
Petechiae	1 (7)	0	0	0
Rash	1 (7)	0	0	0
Sinus tachycardia	1 (7)	0	0	0
Syncope	1 (7)	1 (7)	0	0
Tachycardia	1 (7)	0	0	0
Thrombophlebitis	1 (7)	0	0	0
Transaminases increased	1 (7)	1 (7)	0	0
Headache	0	0	3 (20)	0
Pain in extremity	0	0	3 (20)	0
Anaemia	0	0	2 (13)	2 (13)
Device related infection	0	0	2 (13)	2 (13)
Disease progression	0	0	2 (13)	2 (13)
General physical health deterioration	0	0	2 (13)	1 (7)
Night sweats	0	0	2 (13)	0
Appetite disorder	0	0	1 (7)	0
Atrial fibrillation	0	0	1 (7)	0
Blood creatinine increased	0	0	1 (7)	0
Bone neoplasm	0	0	1 (7)	0
Burns second degree	0	0	1 (7)	0
Cardiac failure	0	0	1 (7)	1 (7)
Conjunctivitis	0	0	1 (7)	0
Decreased appetite	0	0	1 (7)	0
Dysgeusia	0	0	1 (7)	0
Dysphagia	0	0	1 (7)	0
Dysuria	0	0	1 (7)	0
Faeces hard	0	0	1 (7)	0
Febrile infection	0	0	1 (7)	1 (7)
Flank pain	0	0	1 (7)	0
Flatulence	0	0	1 (7)	0
Fungal skin infection	0	0	1 (7)	0
Gingival bleeding	0	0	1 (7)	0
Haematochezia	0	0	1 (7)	1 (7)
Haemoglobin decreased	0	0	1 (7)	1 (7)
Haemoptysis	0	0	1 (7)	1 (7)
Herpes simplex	0	0	1 (7)	0
Hyperglycaemia	0	0	1 (7)	0
Hypotension	0	0	1 (7)	0
Insomnia	0	0	1 (7)	0
Joint injury	0	0	1 (7)	0
Lymphadenitis	0	0	1 (7)	0
Muscle spasms	0	0	1 (7)	0
Non-cardiac chest pain	0	0	1 (7)	0
Oral herpes	0	0	1 (7)	0
Oropharyngeal pain	0	0	1 (7)	0
Phlebitis	0	0	1 (7)	0
Refractoriness to platelet transfusion	0	0	1 (7)	1 (7)
Rhinorrhoea	0	0	1 (7)	0
Sepsis	0	0	1 (7)	1 (7)
Stomatitis	0	0	1 (7)	0
Thermal burn	0	0	1 (7)	0
Thrombocytopenia	0	0	1 (7)	1 (7)
Transfusion related complication	0	0	1 (7)	1 (7)
Vaginal infection	0	0	1 (7)	0
Vestibular disorder	0	0	1 (7)	0
Weight decreased	0	0	1 (7)	0

Table S2. Disease characteristics and outcome of patients with an objective response.

**Variables**	**Responders**		
**Treatment arm**	Placebo	Nintedanib	Placebo
**Sex**	male	female	female
**Age, years**	80	74	81
**Disease status**	Newly diagnosed	r/r	r/r
**Previous lines of therapy**, no	0	1	1
**ECOG PS**	1	0	1
**WBC count**, x 10^3/µL	1.5	16.5	1.9
**PB blasts**, %	41	53	unknown
**BM blasts**, %	80	80	22
**ELN 2010 risk**	Favorable	Intermediate I	Intermediate I
**Initial cytogenetics**	Normal Karyotype	Normal Karyotype	Normal Karyotype
**Initial molecular genetics**	*NPM1*^{mut}	*NPM1*^{mut}, *FLT3*-ITD	n.d.
**Time on treatment,** days	364	211	362
**Time to response**, days	108	57	160
**Response duration**, months	9.4	5.1	9.7
**Survival**, months	16.8	10.4	17.7

Abbreviations: *ECOG*, Eastern Cooperative Oncology Group; *PS*, performance status; *AML*, acute myeloid leukemia; *s-AML*, secondary AML; *WBC*, white blood cells; G/l, giga per liter; *PB*, peripheral blood; *BM*, bone marrow;; *ELN*, European LeukemiaNet; *FLT3-ITD,* internal tandem duplication of the FLT3 gene; *NPM1*, nucleophosmin-1.
